# Is Capitate Shift Reliable as a Single Indicator for Failure of Non-operative Management in Distal Radius Fractures?

**DOI:** 10.7759/cureus.43939

**Published:** 2023-08-22

**Authors:** Liam Donnelly, Ioannis Flogaitis, Akshay Lekhi, Daniel Shaerf

**Affiliations:** 1 Trauma and Orthopaedics, London North West University Healthcare NHS Trust, London, GBR

**Keywords:** cast immobilization, distal radius fracture complications, wrist fractures, distal radius fracture management, distal end radius fracture, carpal bones, capitate

## Abstract

Background

Distal radius fractures (DRFs) are the most commonly treated fracture; however, their treatment remains controversial. There is significant variation in the rate of surgical intervention related to a lack of consensus regarding the displacement threshold for surgery. Although studies have advocated that carpal malalignment is the most important radiographic parameter for surgical correction, it is rarely considered in general clinical practice and remains poorly studied. Recently, capitate shift was identified as the most useful measure of carpal malalignment, and a capitate shift threshold of -5.98 mm was proposed to indicate surgical intervention. This study aimed to investigate if this threshold is associated with the failure of non-operatively managed DRFs and should be used as a threshold for primary surgical intervention.

Methodology

A retrospective analysis was performed of all adult patients who underwent closed manipulation and cast immobilisation for DRFs in a UK district general hospital between September 2021 and February 2022. Capitate shift was measured on initial post-casting radiographs using the validated capitate-to-axis-of-radius distance (CARD) by a junior surgeon. The outcome measure was the failure of conservative management, which was defined as the need for repeat intervention (i.e., cast reapplication or surgical fixation) following closed reduction and cast immobilisation.

Results

A total of 64 patients with 65 DRFs (16 (25%) male, 49 (75%) female) were included in the study. The mean age was 66.6 years (SD = 17.9, 95% CI = 62.2 to 70.9). The mean capitate shift was -1.51 mm (SD = 5.05, 95% CI = -0.28 to -2.73) in all cases (n = 65). The failure rate of DRFs with an ‘unacceptable’ capitate shift (i.e., equal or less than -5.98 mm) compared to those with an ‘acceptable’ capitate shift (i.e., greater than -5.98 mm) was 16.7% versus 3.8% (p = 0.09).

Conclusions

The study concluded that there was no significant association between a capitate shift threshold of -5.98 mm and failure of non-operatively managed DRFs. Given the ease of use and reliability of capitate shift, we advocate for multicentre large cohort studies to identify a threshold for surgical intervention and establish its association with functional outcomes.

## Introduction

Distal radius fractures (DRFs) are the most commonly treated fracture worldwide, with 70,000 cases reported each year in the United Kingdom alone [1]. DRFs have a bimodal distribution, with the first peak in the paediatric population and the second peak in the elderly population, in the latter of whom DRFs represent 18% of all fractures [2]. DRF treatment aims to restore function and minimise complications such as loss of reduction, nerve injury, and pain [3].

Despite the high prevalence of DRFs, optimal treatment remains controversial, with studies reporting a significant variation in the rate of surgical intervention for DRFs [1]. Clinicians typically rely on radiographic parameters to determine if surgical intervention is indicated for displaced DRFs. However, the evidence base for these parameters is inconsistent and varied [4]. Best practice guidelines published by the British Orthopaedic Association and the British Society for Surgery of the Hand in 2018 concluded that there is insufficient evidence to define which of these parameters are important or what the acceptable displacement threshold should be [5]. Therefore, a consensus regarding the displacement threshold for surgical intervention is critical.

A subsequent Delphi study identified carpal malalignment as an important radiographic parameter by 74% of experts [1]. Studies have also suggested that carpal malalignment results in poor functional outcomes by reducing grip strength and range of movement [6,7]. Despite this, carpal malalignment is rarely considered or measured in practice, and there is a distinct lack of evidence to support its use in clinical decision-making [4]. A recent investigation of carpal malalignment by Dias et al. [4] concluded that capitate shift is the most useful measure of carpal malalignment and is strongly associated with dorsal tilt. Statistical analysis of patients with undisplaced or minimally displaced distal radial fractures demonstrated that an acceptable lower limit of capitate shift was -5.98 mm. This correlated with a mean dorsal tilt of -9.1 degrees, which justified the -10 degree threshold for an ‘acceptable’ dorsal tilt recommended by the Delphi panel [1]. This single-centre study aimed to investigate whether a -5.98 mm capitate shift threshold is associated with the failure of non-operatively managed DRFs and should act as a threshold for primary surgical intervention.

## Materials and methods

**Figure 1:**
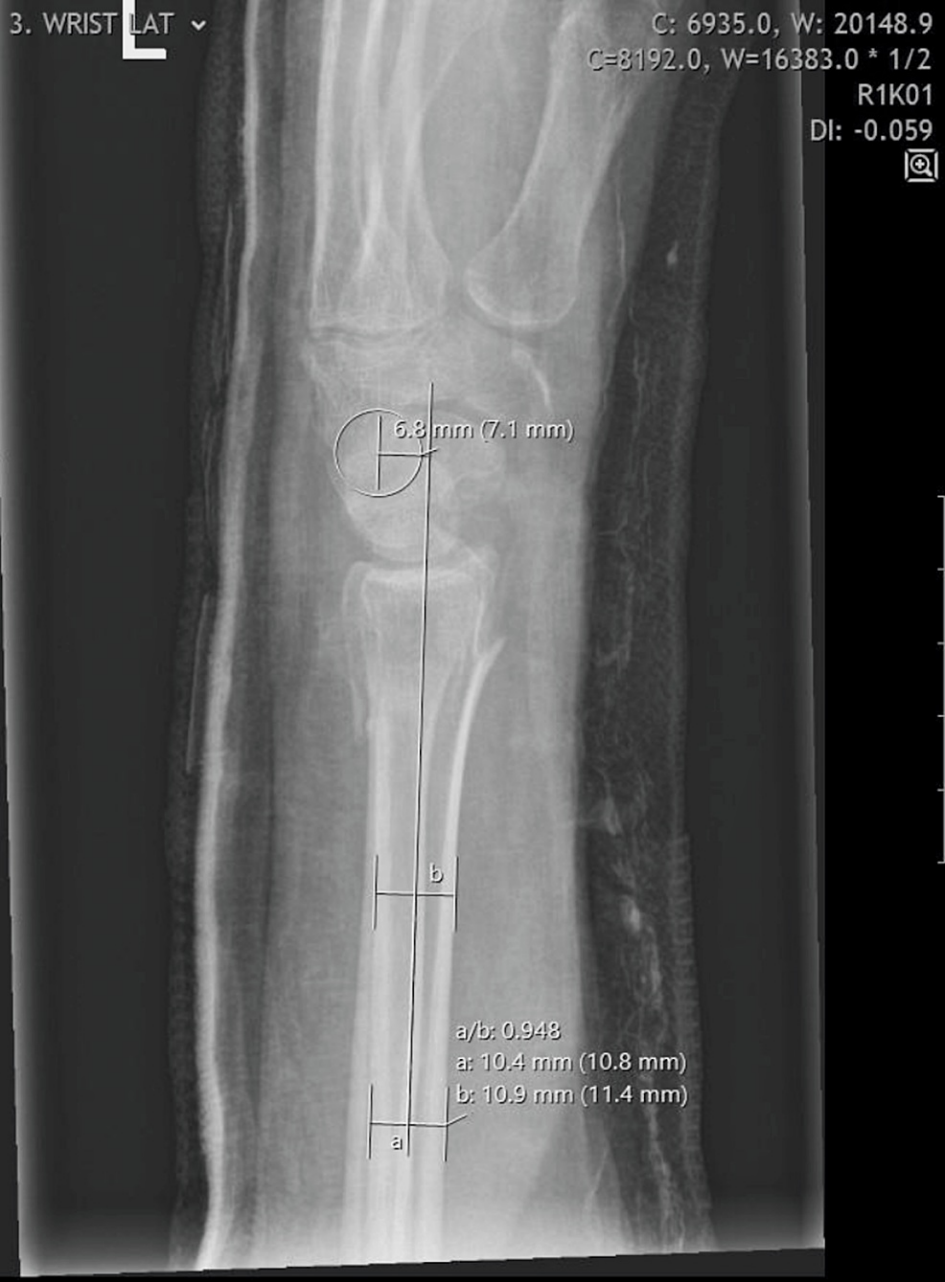
Lateral view radiograph of the left wrist showing measurement of the capitate-to-axis-of-radius distance. The capitate shift measures -6.8 mm on this radiograph.

A retrospective analysis was conducted between September 2021 and February 2022 of all patients aged over 16 years who underwent closed manipulation and cast immobilisation for DRFs in a district general hospital in the United Kingdom. The project was approved by the hospital’s Governance Department. A total of 76 adult patients with 77 DRFs were identified. Demographic data were collected from electronic records. Carpal malalignment was measured on initial post-casting lateral view wrist radiographs using capitate shift as a proxy. Capitate shift measures the shift of the centre of rotation of the capitate from the axis of the radius proximal to the fracture [4]. Capitate shift was measured by one trauma and orthopaedic surgery senior house officer (SHO) using the validated capitate-to-axis-of-radius distance (CARD) (Figure 1) [8]. The CARD is defined as the perpendicular distance from the centre of the head of the capitate to the sagittal axis of the radius proximal to the fracture. The centre of the capitate is identified by placing a circle over the proximal arc of the capitate. The sagittal axis of the radial shaft was determined using the central axis of the radial shaft. This represents the mid-axis of the radius between the dorsal and the volar cortices of the radius [4,8]. The CARD is negative if the capitate lies dorsal to the axis of the radius and positive if it is palmar to this axis [4]. Clinic letters and follow-up radiographs were reviewed to identify any re-displacement requiring cast reapplication or surgical intervention. The outcome measure was the failure of conservative management, which was defined as the need for repeat intervention (i.e., cast reapplication or surgical fixation) following primary closed reduction and cast immobilisation.

Patients were excluded from the study if they had volar displacement (n = 4), declined surgery (n = 3), were not offered surgery due to comorbidities and/or frailty (n = 3), had bone demineralisation preventing accurate capitate shift measurement (n = 1), or had scapholunate widening (n = 1). This yielded a total of 64 patients with 65 DRFs which were included in the study. See Table 1 for demographic data of the included patients.

**Table 1:** Demographic data of the 65 cases. One patient had bilateral fractures.

Characteristics	Data (n = 65)
Mean age, years (range)	66.6 (21–95)
Sex, n (%)	
Male	16 (24.6)
Female	49 (75.4)
Associated ulna fracture, n (%)	
Yes	35 (53.8)
No	30 (46.2)
Complete displacement, n (%)	
Yes	0 (0)
No	65 (100)
Intra-articular fracture, n (%)	
Yes	2 (3.1)
No	63 (96.9)
Failure, n (%)	
Yes	4 (6.2)
No	61 (93.8)

Statistical analysis

A normal probability plot on Google Sheets was created which enabled the analysis and confirmation of the normal distribution of capitate shift values. Descriptive statistics were calculated for the characteristics of the patients and fractures and the radiographic measurements using Google Sheets. Student’s t-test was performed to determine if capitate shift differed between cases which succeeded and failed conservative treatment. Pearson’s chi-square test was performed to assess statistically significant differences between the groups with ‘unacceptable’ and ‘acceptable’ capitate shifts. Statistical significance was set at p-values <0.05.

## Results

**Figure 2:**
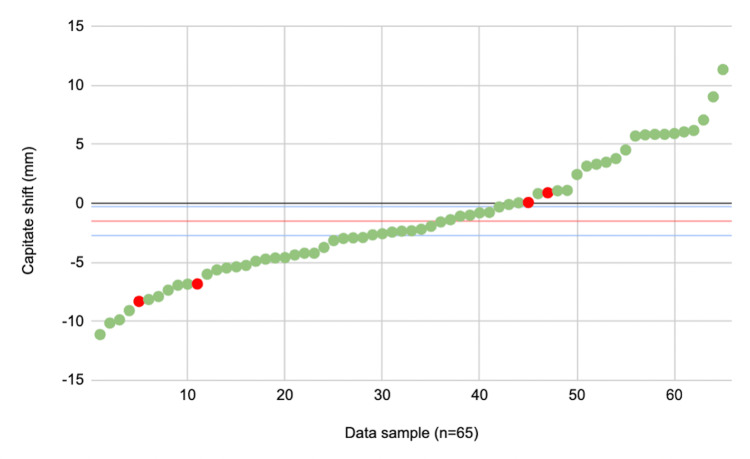
Scatter plot displaying the capitate shift measurements for all DRF cases included in the study (n = 65). Red dots = cases which failed conservative management. Green dots = cases which were successfully treated by conservative management. Red line = mean capitate shift (-1.51 mm). Blue lines = upper (-0.28 mm) and lower (-2.73 mm) 95% confidence intervals. DRF = distal radius fractures

The mean capitate shift was -1.51 mm (SD = 5.05, 95% CI = -0.28 to -2.73) in all cases (n = 65) included in our study (Table 2). The failure rate of conservatively treated DRFs was 6.2% (n = 4) (Figure 2). The mean capitate shifts of successful and failure cases were -1.37 mm (SD = 5.08, 95% CI = -2.65 to -0.10) and -3.55 mm (SD = 4.70, 95% CI = -8.15 to 1.06), respectively. However, these differences were not significantly different (p = 0.41).

**Table 2:** Table displaying the mean capitate shift of DRFs for all cases included in the study (cases which were treated successfully with conservative management, and cases which failed conservative management). A p-value of 0.41 indicates that there was no significant difference in capitate shift measurements between successful and failed cases. DRF = distal radius fractures

Cases	Numbers of DRFs	Mean capitate shift ± SD, mm	P-value
All	65	-1.51 ± 5.05	n/a
Successful	61	-1.37 ± 5.08	0.41
Failure	4	-3.55 ± 4.70	

**Figure 3:**
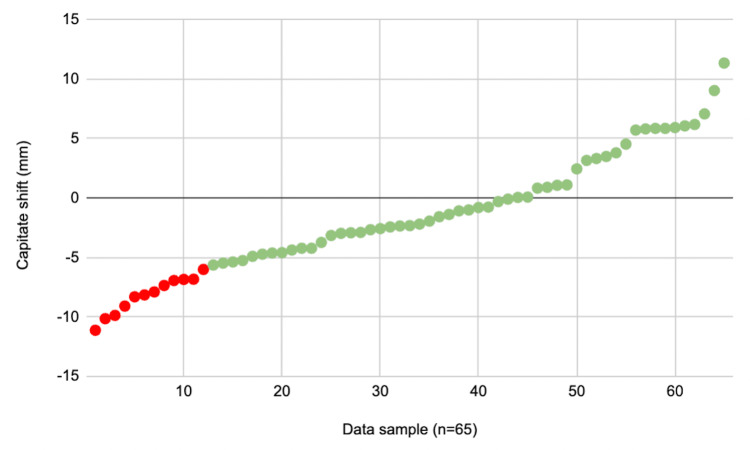
Scatter plot displaying the capitate shift measurements. Red dots = ‘unacceptable’ capitate shift (i.e., equal or less than -5.98 mm). Green dots = ‘acceptable’ capitate shift (i.e., greater than -5.98 mm).

Cases were divided into two groups to assess if a capitate shift threshold of -5.98 mm was associated with the failure of non-operatively managed DRFs. Group 1 (n = 12) comprised cases with an ‘unacceptable’ capitate shift (i.e., equal or less than -5.98 mm), and Group 2 (n = 53) comprised cases with an ‘acceptable’ capitate shift (i.e., greater than -5.98 mm) (Figure 3). The failure rates of Group 1 and Group 2 were 16.7% and 3.8%, respectively, and there was no statistically significant difference (p = 0.09). Similarly, age (p = 0.27), sex ratios (p = 0.48), rate of intra-articular fracture (p = 0.24), and the rate of associated ulna styloid fracture (p = 0.32) did not differ significantly between Group 1 and Group 2 (Table 3).

**Table 3:** Table comparing the characteristics and failure rate between Group 1 and Group 2, including p-value to assess for a statistically significant difference between Group 1 and Group 2.

Characteristics	Group 1 (n = 12)	Group 2 (n = 53)	P-value
Age, mean ± SD, year	64.8 ± 13.4	71.8 ± 18.7	0.27
Sex, n (%)			
Male	2 (16.7)	14 (26.4)	0.48
Female	10 (83.3)	39 (73.6)	
Associated ulna fracture, n (%)			
Yes	8 (66.7)	27 (50.9)	0.32
No	4 (33.3)	26 (49.1)	
Complete displacement, n (%)			
Yes	0 (0)	0 (0)	n/a
No	12 (100)	53 (100)	
Intra-articular fracture, n (%)			
Yes	1 (8.3)	1 (1.9)	0.24
No	11 (91.7)	52 (98.1)	
Failure, n (%)			
Yes	2 (16.7)	2 (3.7)	0.09
No	10 (83.3)	51 (96.2)	

## Discussion

Our study was unable to demonstrate a statistically significant association between a -5.98 mm capitate shift threshold and failure of non-operatively managed DRFs. Nor was there a significant difference in capitate shift between cases with successful versus failed non-operative management. Recent literature has suggested that capitate shift represents an easy, reliable, and reproducible measurement of carpal malalignment, which is the most important radiological indication for surgical intervention for DRFs [4,9].

Traditionally, several other radiographic parameters have been used in the assessments of DRFs to guide treatment choice and predict outcomes [4,9]. A 2018 systematic review identified the most commonly used parameters, namely, radial height, radial inclination, dorsal tilt, ulnar variance, and intra-articular step and gap [5]. However, evidence was insufficient to define which of these parameters were important or what the acceptable displacement threshold should be [5]. The substantial variation in the rate of surgical treatment for DRFs arguably reflects the ambiguity of displacement thresholds for intervention. This ambiguity in the context of the popularisation of surgical treatment for DRFs raises concerns about unwarranted surgical interventions and their associated risks, such as patient safety risks and resource mismanagement [1,10].

A subsequent Delphi study [1] involving national and international experts was performed to identify clinically important radiographic parameters and quantify the displacement thresholds at which surgical intervention should be offered. Expert participants agreed that ulnar variance and dorsal tilt represent the most important extra-articular parameters and step is the most important intra-articular parameter [1]. In addition, 74% of the expert panel highlighted that carpal malalignment represented an important radiographic parameter [4]. Carpal malalignment is defined as the displacement of the longitudinal axis of the capitate either dorsal or volar to the longitudinal axis of the radius [11]. Carpal malalignment following a distal radial fracture occurs due to loss of volar tilt. Change in the orientation of the radial articular surface adversely influences the intercarpal relationship. Loss of volar tilt causes the lunate to dorsiflex with compensatory flexion of the capitate [4].

In comparison to other radiographic parameters, there is a distinct lack of literature discussing carpal malalignment. Many of the Delphi panel experts believed that carpal malalignment resulted in poor functional outcomes; this association is supported by some evidence suggesting diminished grip strength and limited range of movement in cases of malalignment [1,6,7]. Moreover, Ng and McQueen [9] stated that carpal malalignment measurement is more accurate than dorsal tilt as it is less dependent on the orientation of the radiograph. They proposed that carpal malalignment is the most important radiological indication for surgical correction, with measured dorsal tilt being of secondary importance [9]. Nonetheless, carpal malalignment is rarely considered or measured in practice [1].

In a recent investigation of carpal malalignment, Dias et al. [4] concluded that capitate shift is the most useful measure of carpal malalignment. They showed that capitate shift consistently had the strongest relationship with dorsal tilt and was the only radiographic measurement not affected by age or wrist position when compared to radiolunate and capitolunate angles [4]. Undisplaced or minimally displaced DRFs (defined as those with a dorsal tilt up to 0 degrees) had a mean capitate shift of 1.8 mm (99th percentile range = -5.98 to 7.06). Statistical analysis correlated a -5.98 mm capitate shift with a dorsal tilt of -9.1 degrees, which provided support for the -10 degree threshold for the ‘acceptable’ dorsal tilt recommended by the Delphi panel [1]. Based on the findings of Dias et al. [4], this study investigated whether a capitate shift threshold of -5.98 mm is associated with the failure of non-operatively managed DRFs to determine its applicability as a threshold for primary surgical intervention. We found that there was no statistically significant difference in the failure rate of conservatively managed DRFs when using the threshold of -5.98 mm capitate shift. The mean failure rate for Group 1 (‘unacceptable’ capitate shift) and Group 2 (‘acceptable’ capitate shift) was 16.7% and 3.8%, respectively (p = 0.09). We suspect that the small sample size (n = 65) may have precluded statistical significance.

Our capitate shift measurements were consistent with those of Dias et al. [4] who used the mid-axis of the radius rather than the volar cortex of the shaft of the radius and defined the centre of the head of the capitate as a specific point (identified by placing a circle over the proximal arc of the capitate) rather than the axis of the capitate, which is affected by the position of the wrist and was used by previous studies [7,8]. We analysed our patient cohort to identify possible confounding factors responsible for fracture re-displacement. According to the literature, patient age is the factor most consistently associated with an increased risk of re-displacement [12-16]. The ages of our Group 1 (n = 12) and Group 2 (n = 53) participants did not differ significantly, with the mean age being 64.8 and 71.8 years, respectively (p = 0.27). Similarly, the sex ratio of Groups 1 and 2 was comparable (p = 0.48), which nullifies the possible confounding effect of women being at greater risk of re-displacement [16]. Studies have also shown an association between fracture re-displacement and comminution and measurements of displacement, such as ulnar variance; however, we did not assess for these measures in our study [12,15-17]. Notably, intra-articular fractures are not thought to be predictive of re-displacement, although evidence is conflicting; however, they do represent the most significant radiographic predictors of poor functional outcome [15,16]. The rate of intra-articular fractures did not significantly differ between Group 1 and Group 2 (p = 0.24). Moreover, associated ulna styloid fractures were not related to an increased risk of re-displacement and were distributed similarly between Groups 1 and 2, affecting 66.7% and 50.9% of participants, respectively (p = 0.32) [16].

Excluding Dias et al. [4], we are not aware of any other studies that have quantified capitate shift with regard to dorsal tilt or treatment threshold. Before Dias et al. [4], investigations of capitate shift seemed to be visually classified rather than measured [7]. The easy visualisation of the relationship between the capitate and the radius serves as a key advantage of capitate shift over other measurements of carpal malalignment and enables a quick and simple assessment of carpal alignment in clinical situations where it is impractical to measure distances or angles [4]. However, a measurable threshold of capitate shift represents a more accurate and reliable technique to determine if surgical intervention is indicated, thereby reducing the rate of unwarranted surgical interventions.

Limitations of this study include a single-centre data collection in which capitate shift measurements were performed by one orthopaedic SHO without senior review. However, we attempted to mitigate this effect by using the validated CARD measurement tool which has excellent intra- and interobserver reliability and is considered to be unaffected by suboptimal projection [8]. The study is also limited by the inherent selection bias related to retrospective data collection. Our small sample size represents a key limitation. A retrospective study power calculation revealed that 233 participants were required to achieve a study power of 80% (a limit which is considered adequate in the literature). This confirms that our results are subject to type-II error, whereby an inadequate sample size confers an unacceptably high risk of yielding false-negative results, or in this case, a lack of association between the capitate shift threshold and the rate of treatment failure. Therefore, we advocate for larger studies to investigate capitate shift to determine its applicability as a radiographic parameter and to quantify an acceptable displacement threshold for guiding surgical intervention of DRFs. Moreover, this study did not consider the association between capitate shift and functional outcomes, and the authors were unable to identify any prior investigations of capitate shift and its effect on functional outcomes. We believe that a greater understanding of the relationship between capitate shift and functional outcomes would enhance its applicability as a radiographic parameter, and therefore, advocate for further studies.

## Conclusions

The study concluded that there was no significant association between a capitate shift threshold of -5.98 mm and failure of non-operatively managed DRFs. Therefore, there remains no consensus regarding the application of a capitate shift threshold to indicate surgical intervention for DRFs. Given the reliability and reproducibility of capitate shift measurements, we advocate for multicentre large cohort studies to identify a threshold for surgical intervention and establish its association with functional outcomes.

## References

[1] Johnson N, Leighton P, Pailthorpe C, Dias J (2019). Defining displacement thresholds for surgical intervention for distal radius fractures - a Delphi study. PLoS One.

[2] Gutiérrez-Espinoza H, Araya-Quintanilla F, Olguín-Huerta C, Gutiérrez-Monclus R, Valenzuela-Fuenzalida J, Román-Veas J, Campos-Jara C (2022). Effectiveness of surgical versus conservative treatment of distal radius fractures in elderly patients: a systematic review and meta-analysis. Orthop Traumatol Surg Res.

[3] Jamnik AA, Chacon J, Xiao AX, Wagner ER, Gottschalk MB (2022). The effect immobilization mechanisms have on radiographic outcomes and complication rates in the conservative treatment of distal radius fractures: a systematic review. Hand (N Y).

[4] Dias R, Johnson NA, Dias JJ (2020). Prospective investigation of the relationship between dorsal tilt, carpal malalignment, and capitate shift in distal radial fractures. Bone Joint J.

[5] (2023). Best practice for management of distal radial fractures (DRFs). British Orthopaedic Association (BOA), British Society for Surgery of the Hand (BSSH). https://www.bssh.ac.uk/_userfiles/pages/files/professionals/Radius/Blue%20Book%20DRF%20Final%20Document.pdf.

[6] Batra S, Gupta A (2002). The effect of fracture-related factors on the functional outcome at 1 year in distal radius fractures. Injury.

[7] McQueen MM, Hajducka C, Court-Brown CM (1996). Redisplaced unstable fractures of the distal radius: a prospective randomised comparison of four methods of treatment. J Bone Joint Surg Br.

[8] Kuhnel SP, Bigham AT, McMurtry RY, Faber KJ, King GJ, Grewal R (2019). The capitate-to-axis-of-radius distance (CARD): a new radiographic measurement for wrist and carpal alignment in the sagittal plane. J Hand Surg Am.

[9] Ng CY, McQueen MM (2011). What are the radiological predictors of functional outcome following fractures of the distal radius?. J Bone Joint Surg Br.

[10] Walenkamp MM, Mulders MA, Goslings JC, Westert GP, Schep NW (2017). Analysis of variation in the surgical treatment of patients with distal radial fractures in the Netherlands. J Hand Surg Eur Vol.

[11] Taleisnik J, Watson HK (1984). Midcarpal instability caused by malunited fractures of the distal radius. J Hand Surg Am.

[12] Mackenney PJ, McQueen MM, Elton R (2006). Prediction of instability in distal radial fractures. J Bone Joint Surg Am.

[13] Jung HW, Hong H, Jung HJ, Kim JS, Park HY, Bae KH, Jeon IH (2015). Redisplacement of distal radius fracture after initial closed reduction: analysis of prognostic factors. Clin Orthop Surg.

[14] Makhni EC, Ewald TJ, Kelly S, Day CS (2008). Effect of patient age on the radiographic outcomes of distal radius fractures subject to nonoperative treatment. J Hand Surg Am.

[15] Luokkala T, Laitinen MK, Hevonkorpi TP, Raittio L, Mattila VM, Launonen AP (2020). Distal radius fractures in the elderly population. EFORT Open Rev.

[16] Walenkamp MM, Aydin S, Mulders MA, Goslings JC, Schep NW (2016). Predictors of unstable distal radius fractures: a systematic review and meta-analysis. J Hand Surg Eur Vol.

[17] Wadsten MÅ, Sayed-Noor AS, Englund E, Buttazzoni GG, Sjödén GO (2014). Cortical comminution in distal radial fractures can predict the radiological outcome: a cohort multicentre study. Bone Joint J.

